# Insecticidal, Antimalarial, and Antileishmanial Effects of Royal Jelly and Its Three Main Fatty Acids, *trans*-10-Hydroxy-2-decenoic Acid, 10-Hydroxydecanoic Acid, and Sebacic Acid

**DOI:** 10.1155/2022/7425322

**Published:** 2022-01-20

**Authors:** Abeer Mousa Alkhaibari, Abdullah D. Alanazi

**Affiliations:** ^1^Department of Biology, Faculty of Science, University of Tabuk, Tabuk 71491, Saudi Arabia; ^2^Department of Biological Science, Faculty of Science and Humanities, Shaqra University, Ad-Dawadimi 11911, Saudi Arabia

## Abstract

Natural products and their derivatives as an inexpensive, accessible, and useful alternative medicine are broadly applied for the treatment of a wide range of diseases and infectious ones. The present study was designed to evaluate the insecticidal, antimalarial, antileishmanial, and cytotoxic effects of royal jelly and its three main fatty acids (*trans*-10-hydroxy-2-decenoic acid (10-H2DA), 10-hydroxydecanoic acid (10-HDAA), sebacic acid (1,10-decanedioic acid)). Insecticidal activity of RJ and 10-H2DA, 10-HDAA, and sebacic acid was performed against healthy 4th instar larvae at 25 ± 2°C. Antiplasmodial and antileishmanial effects of RJ and 10-H2DA, 10-HDAA, and sebacic acid were also performed against chloroquine-resistant *Plasmodium falciparum* K1-strain and *Leishmania major* amastigotes according to the Malstat method and macrophage model, respectively. In addition, the level of nitric oxide (NO) production in J774-A1 macrophages cells, plasma membrane permeability, and caspase-3-like activity and cytotoxicity effects of RJ and 10-H2DA, 10-HDAA, and sebacic acid against human embryonic kidney 293 (HEK239T cells) were evaluated. Considering the insecticidal activity, the results showed that the lethal concentration 50% value for RJ, 10-H2DA, 10-HDAA, and sebacic acid was 24.6, 31.4, 37.8, and 44.7 *μ*g/mL *μ*g/mL, respectively. RJ, 10-H2DA, 10-HDAA, and sebacic acid showed potent (*P* < 0.0001) antileishmanial effects with IC_50_ values ranging from 2.4 to 8.4 *μ*g/mL. Various concentrations of RJ, 10-H2DA, 10-HDAA, and sebacic acid significantly (*P* < 0.05) increased the production of NO, plasma membrane permeability, and caspase-3-like activity level as a dose-dependent response. Considering the cytotoxicity, SIs > 10 of these compounds exhibited their specificity to parasites and safety against human HEK239T normal cells. The results of the present investigation revealed the promising insecticidal, antimalarial, and antileishmanial effects of RJ and its three main fatty acids (10-H2DA, 10-HDAA, and sebacic acid). However, more studies are required to confirm the mechanisms of action mode of these compounds as well as their efficacy in animal models and clinical settings.

## 1. Introduction

According to the World Health Organization (WHO) reports, vector-borne diseases account for nearly 17% of all infectious diseases, with a mortality rate of 700,000 deaths each year [1]. *Aedes aegypti* is considered as the principal vector recognized to transmit a number of important vector-borne infections such as malaria, dengue, chikungunya, and Zika [2, 3]. Today, one of the most important approaches for controlling insects is the use of insecticides (e.g., diflubenzuron, alpha-cypermethrin, malathion, and deltamethrin) [4, 5]. The current studies demonstrated that the too much and persistent use of chemical and synthetic insecticides has reduced their efficacy and raised various ecological concerns such as emerging of drug resistance to insecticides, ecological imbalance, and outcomes to animals [6, 7]. Accordingly, these problems and limitations have led researchers to pay more attention to finding novel insecticides with high efficacy and eco-friendly properties around the world.

Malaria is a serious vector-borne infection, which causes about 430,000 deaths yearly, generally in African children [8]. Among the *Plasmodium* species in human, *Plasmodium vivax* and *P. falciparum* are well known as the most common and deadly *Plasmodium* species, respectively [9, 10]. In recent decades, there have been numerous reports on side effects as well as drug resistance of *Plasmodium* spp. (mainly *P. falciparum* malaria) to current antimalarial drugs [11]. So finding and introducing a new antimalarial drug especially from natural resources with the lower toxicity and the highest performance is necessary for malaria treatment.

Leishmaniasis as a neglected tropical infection is caused by the parasitic species *Leishmania,* which infects about 12 million people each year in 98 countries worldwide [12]. The disease is observed in humans in different forms, including cutaneous leishmaniasis, cutaneous-mucosal leishmaniasis, and visceral leishmaniasis [13]. In chemotherapy as the most important method of treating leishmaniasis, various drugs such as meglumine antimoniate and Pentostam are used; however, the previous studies have reported that the majority of the synthetic antileishmanial drugs have been associated with some disadvantages and limitations such as emerging drug resistance and adverse side effects [14, 15].

From a long time ago, natural products have been well known as a valuable and effective resource for treating a broad spectrum of diseases, including cardiovascular, cancers, and neurological [16]. Among the natural products, beehive derivatives and products including royal jelly, honey, and propolis are considered as one of the best health-promoting products [17]. Royal jelly (RJ) is a gelatinous material secreted from the apical glands of young nurse honey bees with various pharmacological and biological benefits such as anticancer, antioxidant, anti-inflammatory, and antimicrobial [18–20]. Considering the compounds in RJ, previous studies have demonstrated that the main composition of RJ is water (50–60%), proteins (18%), carbohydrates (7–18%), and lipids (3–8%), respectively [21]. The lipid composition of RJ consists of 80–85% fatty acids, composed of proteins that are attributed to its biological properties [22]. Reviews showed that the major fatty acids present in RJ are (i) *trans*-10-hydroxy-2-decenoic acid (10-H2DA), an unsaturated hydroxyl fatty acid which consists of >50% of the free FAs; (ii) 10-hydroxydecanoic acid (10-HDAA), as a saturated hydroxyl fatty acid, 10-HDAA; (iii) sebacic acid (SEA, 1,10-decanedioic acid), a dicarboxylic fatty acid, comprising 3.3% of the total acids in RJ [23, 24].

Based on what has been said about the biological and pharmacological activities of RJ and also the limitations, problems, and side effects of the current insecticidal, antileishmanial, and antimalarial agents, in this study, we decided to assess the insecticidal, antiplasmodial, antileishmanial, and cytotoxic effects of RJ and its three main fatty acids (10-H2DA, 10-HDAA, and sebacic acid).

## 2. Materials and Methods

### 2.1. Royal Jelly

Royal jelly materials were obtained from Langstroth hives containing colonies of the *A. mellifera* grown in Tabuk, Saudi Arabia, from June 2021. Samples were dissolved with normal saline and filtered under a vacuum by means of filter paper (Whatman membrane, England). Samples (50 mg) were diluted in distilled water and then ultrasonicated for 1 h. In the next step, the sample solution was centrifuged at 6000 rpm for 10 minutes, whereas the upper phase was applied as the sample solution and was stored at −20°C until testing.

### 2.2. Secondary Metabolites Contents

#### 2.2.1. Total Phenol Content

The total phenol content in the RJ sample was assessed based on the technique explained by Singleton et al. [25]. To do this, 50 *μ*L of RJ solution (50 mg/mL) was added to 250 *μ*L of Folin–Ciocalteu's reagent (0.2 N) for 6 min, and then 0.2 mL of sodium carbonate (7.5%) was put into the tested tubes. After 120 min incubation at room temperature, the absorbance of suspension was determined at 760 nm. Distilled water was used as the blank solution. Total phenol content was reported as mg gallic acid equivalents (GAE)/g.

#### 2.2.2. Total Flavonoid Content

The determination of flavonoid content was performed according to the method explained by El-Guendouz et al. In this way, 200 *μ*L of RJ solution was poured into the tubes with 200 *μ*L aluminum chloride (20%). The tubes were then incubated at room temperature for 60 min, and their absorbance was determined at 420 nm [26]. The total flavonoid content was reported as milligram of quercetin equivalents per gram of RJ (mg QE/g RJ).

#### 2.2.3. Total Protein Content

Bradford technique was used to determine the protein content. Briefly, the RJ sample (200 mg) was added to a tube containing 10 mL methanol/water (50/50; v/v); the suspension was sonicated for 1 h. In the next step, the pH of the suspension was adjusted to 2.5 with phosphoric acid and was then diluted 10 times. Next, 5 mL of Bio-Rad reagent was mixed with 250 *μ*L of RJ solutions, and the absorbance of the mixture was determined at 595 nm. The total protein content was reported as a percentage by means of the bovine serum albumin standard curve [27].

### 2.3. Chemicals

*trans*-10-hydroxy-2-decenoic acid, 10-hydroxydecanoic acid, sebacic acid (1,10-decanedioic acid), and MTT (3-(4,5-dimethylthiazol-2-yl)-2,5-diphenyltetrazolium bromide) powder in high purity were purchased from Sigma-Aldrich (Germany).

### 2.4. Insecticidal Activity

*Ae. aegypti* eggs were provided from the Department of Biology, Faculty of Science, University of Tabuk, Saudi Arabia. The insecticidal effects of RJ, 10-H2DA, 10-HDAA, and sebacic acid were carried out based on the method described by Huong et al. [28]. To do this, various concentrations of RJ, 10-H2DA, 10-HDAA, and sebacic acid (12.5, 25, 50, and 100 *μ*g/mL) were put in a 500 mL beaker, and 150 mL water was added with 20 healthy 4th instar larvae at 25 ± 2°C. Larva mortality was determined after 24 h of incubation; lethal concentration 50% (LC_50_) was calculated via the Probit test in SPSS software for RJ, 10-H2DA, 10-HDAA, and sebacic acid. All tests were carried out in triplicate, and during experiments, no nutritional complement was added, whereas DMSO was considered as the control group.

### 2.5. Antiplasmodial Activity

The Malstat method was used to determine the antiplasmodial effects of different concentrations of RJ, 10-H2DA, 10-HDAA, and sebacic acid (12.5, 25, 50, and 100 *μ*g/mL) against *P. falciparum* K1-strain [29]. Parasites were firstly incubated with the human erythrocytes (red blood cells, RBC) in RPMI-1640 medium improved with 10% human serum at 37°C with low oxygen atmosphere (3% O_2_, 4% CO_2,_ and 93% N_2_). The infected human RBC (0.2 mL, 1% parasitaemia, and 2% hematocrit) were treated with various concentrations of RJ, 10-H2DA, 10-HDAA, and sebacic acid in each well and were incubated for 3 days. Then, tested plates were frozen at −20°C. In the next step, in a new plate, 0.1 mL of Malstat reagent was mixed with 0.02 mL of suspension of hemolysed parasite and incubated for 15 min at 21°C. After this time, 0.02 mL of NBT/PES solution was added to the plates and was incubated again for 120 min in the dark. Finally, the absorbance of each well was recorded at 655 nm with the ELISA reader. The 50% inhibitory concentrations (IC_50_) were also calculated via the Probit test in SPSS software.

### 2.6. Antileishmanial Effects

*L. major* promastigotes (MRHO/IR/75/ER) and murine macrophage cells (J774-A1) were cultured at RPMI 1640 complemented with 15% heat-inactivated fetal calf serum (FCS), streptomycin (100 *μ*g/mL), and penicillin (200  IU/mL) and at Dulbecco's modified eagle's medium (DMEM) improved with 10% FCS at 37°C in 5% CO_2_. Firstly, J774-A1 cells (5 × 10^5^ cells/mL) were transferred in sterile 6-cell plates (with 1 cm^2^ coverslip implanted on their floor) and incubator at 37°C for 24 hours with 5% CO_2_ to adhere to macrophages. After 24 hours, the plates were removed from the incubator and washed with sterile warm saline phosphate buffer. Then, 1 mL of *L. major* promastigotes (5 × 10^6^) in the stationary phase was added to plates and kept warm at 37°C for 4 hours; then, the wells were washed with RPMI1640 medium to remove free promastigotes. In the next step, 1 mL of RPMI1640 medium containing different concentrations of RJ, 10-H2DA, 10-HDAA, and sebacic acid (1, 2, 4, and 8 *μ*g/mL) and MA was added to the wells and was incubated for 48 hours. The slides were then fixed with methanol and stained with Gamisa dye diluted with water in a ratio of 1 : 10. The results were estimated by calculating the mean number of amastigotes inside 100 macrophages. The IC_50_ values were also calculated via the Probit test in SPSS software. Examinations were carried out in triplicate [30].

### 2.7. Plasma Membrane Permeability

In order to determine the plasma membrane permeability, *L. major* (1 × 10^6^ cells/mL) were incubated with different concentrations of RJ, 10-H2DA, 10-HDAA, and sebacic acid (12.5, 25, 50, and 100 *μ*g/mL) and then Sytox Green stain was applied based on the kit instructions. Nontreated parasites and those treated with 2.5% of Triton X-100 (Sigma-Aldrich) were determined as the negative and positive control, respectively. The plasma membrane permeability was calculated by means of a microplate reader (BMG Labtech, Germany) for 4 h [30].

### 2.8. Effect on Nitric Oxide (NO) Production

The effect of RJ, 10-H2DA, 10-HDAA, and sebacic acid on the NO release of macrophage cells was performed via Griess reaction for nitrites. In summary, macrophage cells (1 × 10^6^ cells/mL) were exposed with various concentrations of RJ, 10-H2DA, 10-HDAA, and sebacic acid (1, 2, and 4 *μ*g/mL) for 72 h. Then, 0.1 mL of collected supernatants was transferred into a 96-well microplate, and 60 *μ*L of Griess reagents A and B was put within each well. Lastly, the production of NO was determined by reading the plates at 540 nm in an ELISA reader (BioTek-ELX800) [30].

### 2.9. Evaluating the Caspase-3-Like Activity of Extract-Treated Promastigotes

Caspase-3-like activity of *L. major* promastigotes treated with RJ, 10-H2DA, 10-HDAA, and sebacic acid was assessed through the colorimetric protease (Sigma, Germany) technique according to the manufacturer guidelines. In this way, the caspase-3-like activity level was measured based on the rate of color spectrophotometric produced through the release of a molecule (pNA attached to the substrate) under the enzyme caspase-3 activity. Briefly, the promastigotes (10^6^) were incubated with RJ, 10-H2DA, 10-HDAA, and sebacic acid for 48 h and were centrifuged at 700 rpm for 5 minutes at 4°C. In the next step, the cell residue was lysed, and the cell lysate was centrifuged again at 20,000 rpm for 10 minutes. Lastly, supernatant of reaction (5 *μ*L) was added to the 85 *μ*L of buffer, and 10 *μ*L of caspase 3 (pNA-DEVD-Ac) solution and the mixture was incubated for 120 min at 37°C. The caspase-3-like activity was determined through the light absorption at 405 nm with the ELISA reader [29].

### 2.10. Cytotoxicity Effects

The cytotoxic effect of RJ, 10-H2DA, 10-HDAA, and sebacic acid against human embryonic kidney 293 (HEK239T cells) was evaluated using the colorimetric MTT (3-(4,5-dimethylthiazol-2-yl)-2,5-diphenyltetrazolium bromide) assay according to the method described elsewhere [30]. HEK239T cells were cultured in DMEM, supplemented with 10% fetal bovine serum and streptomycin (100 *μ*g/mL) and penicillin (200 IU/mL). Next, HEK239T cells (5 × 10^4^/mL) were treated with RJ, 10-H2DA, 10-HDAA, and sebacic acid (12.5, 25, 50, 100, and 200 *μ*g/mL) for 48 h in microplates at 37°C with 5% CO_2_. The 50% cytotoxic concentrations (CC_50_ values) were calculated by means of the Probit test in SPSS software. Selectivity indices (CC_50_/IC_50_) were also recorded for each tested drug [27].

### 2.11. Statistical Analysis

The statistical analysis was performed by the SPSS statistical package, version 22.0 (SPSS, Inc.). To compare the results among tested groups, we applied the unpaired samples *t*-test, one-way analysis of variance (ANOVA), and Dunnett's test. *P* < 0.05 was measured statistically significant.

## 3. Results

### 3.1. Secondary Metabolites Contents of RJ

The findings of the secondary metabolites analysis of RJ displayed that total phenolic and flavonoid content were 83.6 ± 0.31(mg GEA/g DW) and 1.78 ± 0.023 (mg QE/g DW), respectively; the results also showed that the total protein content of the RJ sample was 11.3% (Table 1).

### 3.2. Insecticidal Effects

Table 2 revealed that the larvicidal effects of RJ, 10-H2DA, 10-HDAA, and sebacic acid were remarkable (*P* < 0.001) against *Ae. aegypti* larva. The results showed that among the tested compounds sebacic acid and RJ displayed the lowest and highest larvicidal effects against *Ae. aegypti* larva with the LC_50_ of 44.7 and 24.6 *μ*g/mL, respectively.

### 3.3. Antimalarial Activity

The obtained findings demonstrated that RJ, 10-H2DA, 10-HDAA, and sebacic acid had considerable (*P* < 0.001) antiplasmodial effects against *P. falciparum*. The IC_50_ value for RJ was 7.62 *μ*g/mL, while the IC_50_ value for 10-H2DA, 10-HDAA, and sebacic acid was 2.41, 2.65, and 3.1 *μ*g/mL, respectively (Table 3), indicating that the lowest and highest antimalarial activities were observed in RJ and 10-H2DA, respectively.

### 3.4. Antileishmanial Effects

Our findings also exhibited that RJ, 10-H2DA, 10-HDAA, and sebacic acid had considerable (*P* < 0.001) antileishmanial effects against intracellular amastigotes of *L. major*. The IC_50_ value for RJ was 8.14 *μ*g/mL, while the IC_50_ value for 10-H2DA, 10-HDAA, and sebacic acid was 3.8, 3.77, and 4.13 *μ*g/mL, respectively (Table 4). These findings indicated that although all tested compounds showed higher antileishmanial effects against intracellular amastigotes of *L. major*, the lowest and highest antileishmanial activities were observed in RJ and 10-H2DA, respectively.

### 3.5. Cytotoxicity Effects

Considering the cytotoxicity effects of RJ, 10-H2DA, 10-HDAA, and sebacic acid, the obtained results of the MTT assay demonstrated that CC_50_ value for RJ, 10-H2DA, 10-HDAA, and sebacic acid was 117.3, 74.4, 81.3, and 92.6 *μ*g/mL; subsequently, their SI > 10 of these compound exhibited their specificity to parasites and safety against human HEK239T normal cells (Table 5), indicating that among the tested compounds the lowest and highest cytotoxic effects were observed in RJ and sebacic acid, with the SI value of 13.9 and 22.4, respectively.

### 3.6. The Effect on the Plasma Membrane Permeability

**Figure 1:**
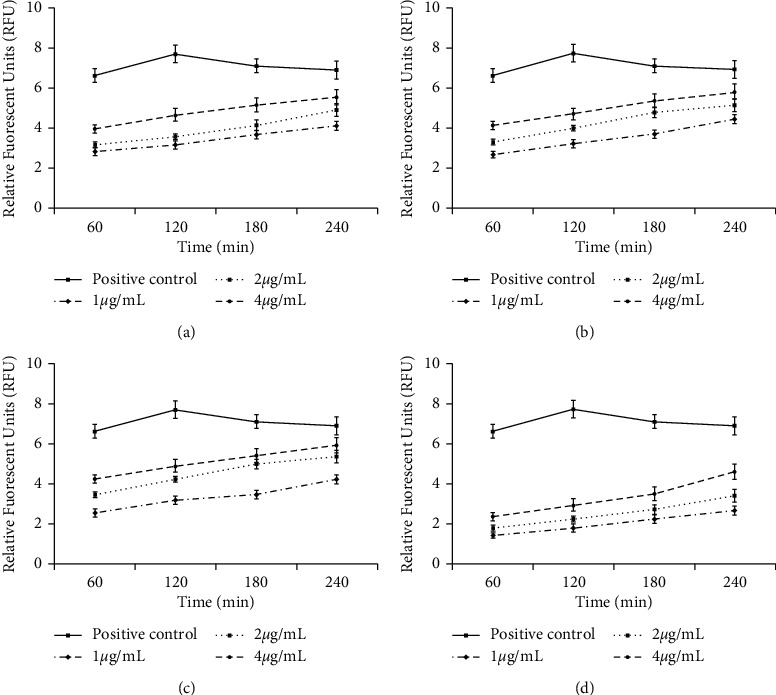
The plasma membrane permeability of the *Leishmania major* promastigotes treated with RJ (a), 10-H2DA (b), 10-HDAA (c), and sebacic acid (d). The results exhibited of relative fluorescence units revealed that the promastigotes treated with RJ, 10-H2DA, 10-HDAA, and sebacic acid as a dose-dependent response changed the permeability of plasma membrane by Sytox Green. Data are presented as the mean ± SD (*n* = 3).

Here, we evaluated the plasma membrane permeability of the *L. major* promastigotes treated with RJ, 10-H2DA, 10-HDAA, and sebacic acid. The results of relative fluorescence units revealed that the promastigotes treated with RJ, 10-H2DA, 10-HDAA, and sebacic acid as a dose-dependent response changed the permeability of plasma membrane by Sytox Green (Figure 1).

### 3.7. Evaluating NO Production

**Figure 2:**
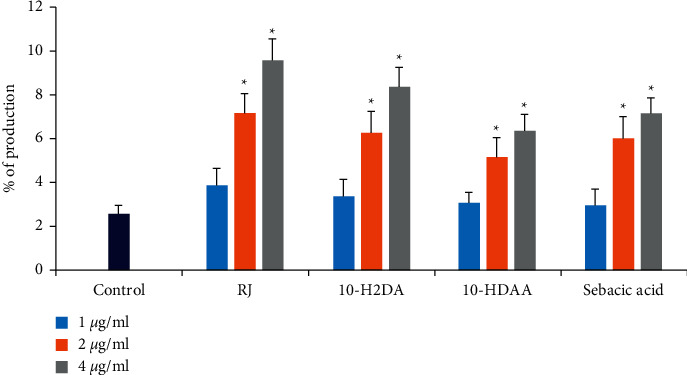
Comparison of NO production in J774-A1 macrophage cells after treatment with various concentrations of royal jelly and its three main fatty acids (*trans*-10-hydroxy-2-decenoic acid (10-H2DA), 10-hydroxydecanoic acid (10-HDAA), and sebacic acid). Data are presented as the mean ± SD (*n* = 3). ^*∗*^*P* < 0.05 shows that the difference was statistically significant in comparison with control.

As shown in Figure 2, various concentrations of RJ, 10-H2DA, 10-HDAA, and sebacic acid (1, 2, and 4 *μ*g/mL) significantly (*P* < 0.05) increased the production of NO as a dose-dependent pattern in comparison to the nontreated macrophage cells as a dose-dependent response. The results also showed that, among the tested compounds, RJ and 10-H2DA displayed the highest stimulation of NO production.

### 3.8. Effect on the Caspase-3-Like Activity

**Figure 3:**
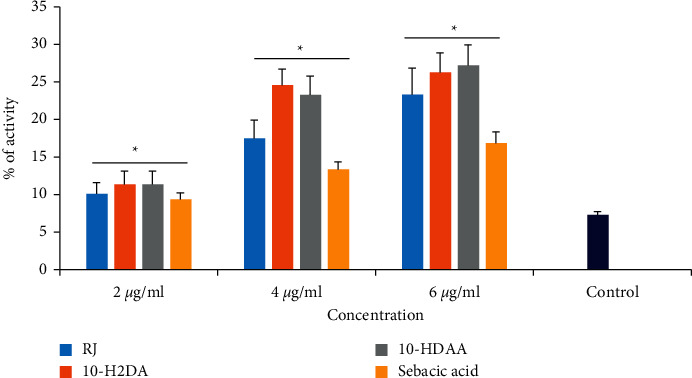
The caspase-3-like activity of promastigotes treated with RJ, 10-H2DA, 10-HDAA, and sebacic acid using the colorimetric protease methods. The results exhibited that RJ, 10-H2DA, 10-HDAA, and sebacic acid significantly induced caspase-3 activation as a dose-dependent response. Data are presented as the mean ± SD (*n* = 3). ^*∗*^*P* < 0.05 shows that the difference was statistically significant in comparison with control.

The caspase-3-like activity of parasites treated with RJ, 10-H2DA, 10-HDAA, and sebacic acid was assessed through the colorimetric protease. The results exhibited that RJ, 10-H2DA, 10-HDAA, and sebacic acid significantly induced caspase-3 activation as a dose-dependent response ranging from 9.4 to 27.2% in comparison with the control (Figure 3). Based on the obtained results, among the tested compounds, 10-H2DA and 10-HDAA displayed the maximum induction of caspase-3 activity.

## 4. Discussion

From the last centuries, natural products have been well known as valuable resources of bioactive and useful ingredients for medical, industrial, and agricultural purposes [31]. In recent years, natural products have been evaluated for several procedures in the pest control insecticidal, ovicidal, and evaluation as a repellent [32–34]. The present study aimed to assess the insecticidal, antiplasmodial, antileishmanial, and cytotoxic effects of RJ and its three fatty acids (10-H2DA, 10-HDAA, and sebacic acid). We found that sebacic acid and RJ displayed the lowest and highest larvicidal effects against *Ae. aegypti* larva, respectively. At present, there is no specific criterion in the recommendations of the WHO for estimating the larvicidal activity of natural products, but several studies have reported some criteria to demonstrate the efficacy of insecticides derived from natural products [35, 36]. For example, Komalamisra et al. [37] have revealed that natural products with the LC_50_ value of less than 50 mg/L are promising and active, while the products with the LC_50_ values between 50 to 100 mg/L were moderately active. In addition, natural products with the LC_50_ value between 100 and 750 mg/L and >750 mg/L were effective and inactive, respectively. Another investigation conducted by Kiran et al. [38] reported that natural products with the LC_50_ less than 100 mg/L are considered as a potent larvicidal effect. However, these criteria are depended on some factors such as exposure time and the source of larvae, which can affect the LC_50_ values of tested natural products [39]. Hence, our results exhibited the relevant and promising insecticidal effects of RJ, 10-H2DA, 10-HDAA, and sebacic acid, according to the criterion reported by Komalamisra et al. [37] and Kiran et al. [38].

Our findings demonstrated that RJ, 10-H2DA, 10-HDAA, and sebacic acid had considerable (*P* < 0.001) antiplasmodial effects against *P. falciparum* with the IC_50_ values 7.62, 2.41, 2.65, and 3.1 *μ*g/mL, respectively. In addition, we found that RJ, 10-H2DA, 10-HDAA, and sebacic acid had considerable (*P* < 0.001) antileishmanial effects against the intracellular amastigotes of *L. major* with the IC_50_ value 8.14, 3.8, 3.77, and 4.13 *μ*g/mL, respectively. Today, there has been rising attention in evaluating the pharmacological and chemical properties of bee-related products as a substitute antiparasitic treatment [40]. Previously, it has been proven that bee-related products such as honey, propolis, bee pollen, and bee venom are widely used as herbal therapies for treating various infectious diseases in various parts of the world [41]. Several investigations have shown the promising *in vitro* and/or *in vivo* antiparasitic effects of bee-related products against a wide range of protozoa and helminths such as *Schistosoma* spp.*, Trypanosoma* spp*., Leishmania* spp*.,* and *Plasmodium* spp. [42]. Considering the antimicrobial mechanisms of bee products, previous studies displayed that these compounds have several antimicrobial mechanisms such as (i) disruption of cell wall, (ii) stimulation of macrophages via the production of reactive oxygen species (ROS) and nitrogen metabolites, (iii) promoting the activation of host immune responses by stimulating the production of some cytokines, and (iv) the induction of apoptosis-like mechanisms [43–50].

We found that the10-H2DA, 10-HDAA, and sebacic acid as the major fatty acids present in RJ had potent insecticidal, antileishmanial, and antiplasmodial effects. Reviews have previously demonstrated that fatty acids demonstrated their antimicrobial mechanisms through suppressing the cellular energy production, inhibition of the DNA/RNA replication, inhibition of enzyme activity, nutrient uptake disorders, generation of peroxidation, and autooxidation degradation products, as well as cytoplasmic membrane disruption [51, 52]. Therefore, it can be suggested that the insecticidal, antiplasmodial, and antileishmanial effects of RJ are due to the existence of these constituents in RJ.

Nowadays, it has been demonstrated that NO, which is produced by a number of immune cells, plays a critical role in the immune-mediated response for eliminating intracellular pathogens [53]. We reported that RJ, 10-H2DA, 10-HDAA, and sebacic acid (1, 2, and 4 *μ*g/mL) significantly (*P* < 0.05) increased the production of NO as a dose-dependent pattern in comparison to the nontreated macrophage cells. These results suggest that although RJ, 10-H2DA, 10-HDAA, and sebacic acid triggered the NO production as an important intracellular antimicrobial mechanism, supplementary surveys and analyses are required to evaluate the importance of NO and eliminate other factors.

Previous studies have exhibited that the rupture plasma membrane is one of the key action modes to inhibit the growth of intracellular pathogens [31, 40]. We found that the promastigotes treated with RJ, 10-H2DA, 10-HDAA, and sebacic acid changed the permeability of the plasma membrane by Sytox Green as a dose-dependent response.

Apoptosis is one of the important processes that basically links pathogen survival to its ability to induce controlled death [43]. Among the caspases, as the main mediators of apoptosis, caspase-3 is considered one of the key caspases that predominantly triggered death protease and successively prompt cell death [45]. Here, we found that RJ, 10-H2DA, 10-HDAA, and sebacic acid significantly induced caspase-3 activation as a dose-dependent response ranging from 9.4 to 27.2% in comparison with the control. Considering the cytotoxicity effects of RJ, 10-H2DA, 10-HDAA, and sebacic acid, the obtained results of the MTT assay demonstrated that the CC_50_ value for RJ, 10-H2DA, 10-HDAA, and sebacic acid was 117.3, 74.4, 81.3, and 92.6, respectively; subsequently, their SI > 10 of these compound exhibited their specificity to parasites and safety against human HEK239T normal cells.

## 5. Conclusion

The results of the present investigation revealed the promising insecticidal, antiplasmodial, and antileishmanial effects of RJ and its three main fatty acids (10-H2DA, 10-HDAA, and sebacic acid) against healthy 4th instar larvae of *Ae. aegypti*, chloroquine-resistant *P. falciparum* K1-strain, and *L. major* amastigotes, respectively. Although the main mechanisms of action in these natural products are clearly understood, our study revealed that RJ, 10-H2DA, 10-HDAA, and sebacic acid displayed their antimicrobial mechanism through the plasma membrane permeability, triggering the NO production, and the induction of apoptosis. More studies are required to confirm the efficacy of these compounds, especially in animal models and clinical settings.

## Figures and Tables

**Table 1:** The results of measurement of the secondary metabolites contents of royal jelly.

Total content	Test	Amount

Phenolic	Folin–Ciocalteu's reagent colorimetric	83.6 ± 0.31 mg GEA/g DW
Flavonoids	Aluminum chloride (AlCl_3_ 2%) colorimetric	1.78 ± 0.023 mg QE/g DW
Protein	Bradford method	11.3%

**Table 2:** Larvicidal activity of royal jelly and its three main fatty acids (*trans*-10-hydroxy-2-decenoic acid (10-H2DA), 10-hydroxydecanoic acid (10-HDAA), and sebacic acid (1,10-decanedioic acid)) against *Ae. aegypti* larva.

Drug	Insecticidal activity LC_50_ (*μ*g/mL)

Royal jelly	24.6
10-H2DA	31.4
10-HDAA	37.8
Sebacic acid	44.7

**Table 3:** Antimalarial activity of royal jelly and its three main fatty acids (*trans*-10-hydroxy-2-decenoic acid (10-H2DA), 10-hydroxydecanoic acid (10-HDAA),and sebacic acid (1,10-decanedioic acid)) against *P. falciparum* K1-strain.

Drug	Antimalarial activity IC_50_ (*μ*g/mL)

Royal jelly	7.62 ± 0.65
10-H2DA	2.41 ± 0.155
10-HDAA	2.65 ± 0.182
Sebacic acid	3.1 ± 0.213
Chloroquine sulphate	0.53 ± 0.213

Data are presented as the mean ± SD.

**Table 4:** Antileishmanial activity of royal jelly and its three main fatty acids (*trans*-10-hydroxy-2-decenoic acid (10-H2DA), 10-hydroxydecanoic acid (10-HDAA), and sebacic acid (1,10-decanedioic acid)) against intracellular amastigotes of *Leishmania major*.

Drug	Antileishmanial effect IC_50_ (*μ*g/mL)

Royal jelly	8.4 ± 0.74
10-H2DA	3.8 ± 0.22
10-HDAA	3.77 ± 0.46
Sebacic acid	4.13 ± 0.51
Meglumine antimoniate	13.6 ± 1.15

Data are presented as the mean ± SD.

**Table 5:** Cytotoxic effects of royal jelly and its three main fatty acids (*trans*-10-hydroxy-2-decenoic acid (10-H2DA), 10-hydroxydecanoic acid (10-HDAA), and sebacic acid (1,10-decanedioic acid)) and their selectivity index (SI) against human HEK239T normal cells.

Drug	Cytotoxicity effect CC_50_ (*μ*g/mL)	SI

Royal jelly	117.3	13.9
10-H2DA	74.4	19.5
10-HDAA	81.3	21.5
Sebacic acid	92.6	22.4

Data are presented as the mean ± SD.

## Data Availability

All data generated or analyzed during this study are included in this published paper.
